# Energy Allocation for LoRaWAN Nodes with Multi-Source Energy Harvesting

**DOI:** 10.3390/s21082874

**Published:** 2021-04-19

**Authors:** Philip-Dylan Gleonec, Jeremy Ardouin, Matthieu Gautier, Olivier Berder

**Affiliations:** 1 Wi6Labs, F-35510 Cesson-Sévigné, France; philip-dylan@gleonec.bzh (P.-D.G.); jeremy.ardouin@wi6labs.com (J.A.); 2 University Rennes, CNRS, IRISA, 22300 Lannion, France; olivier.berder@irisa.fr

**Keywords:** energy-efficient IoT devices, energy harvesting, low-power communications

## Abstract

Many connected devices are expected to be deployed during the next few years. Energy harvesting appears to be a good solution to power these devices but is not a reliable power source due to the time-varying nature of most energy sources. It is possible to harvest energy from multiple energy sources to tackle this problem, thus increasing the amount and the consistency of harvested energy. Additionally, a power management system can be implemented to compute how much energy can be consumed and to allocate this energy to multiple tasks, thus adapting the device quality of service to its energy capabilities. The goal is to maximize the amount of measured and transmitted data while avoiding power failures as much as possible. For this purpose, an industrial sensor node platform was extended with a multi-source energy-harvesting circuit and programmed with a novel energy-allocation system for multi-task devices. In this paper, a multi-source energy-harvesting LoRaWAN node is proposed and optimal energy allocation is proposed when the node runs different sensing tasks. The presented hardware platform was built with off-the-shelf components, and the proposed power management system was implemented on this platform. An experimental validation on a real LoRaWAN network shows that a gain of 51% transmitted messages and 62% executed sensing tasks can be achieved with the multi-source energy-harvesting and power-management system, compared to a single-source system.

## 1. Introduction

The IoT (Internet of Things) has become an important research area in recent years for both the academic and industrial communities, leading to the development of multiple technologies dedicated to this market. LoRaWAN [1] (Long-Range Wide-Area Network), for instance, which uses the LoRa [2] (Long-Range) modulation, enables long-range communication with limited power consumption. As more IoT nodes are deployed, the way they are powered becomes a critical issue. In particular, using non-rechargeable batteries for several billion devices [3] would create a significant amount of chemical waste. Moreover, the use of rechargeable batteries is not always feasible as recharging those would increase the maintenance cost of the network or is even impossible if the IoT node is deployed in a harsh area. Thus, using energy-harvesting technologies can reduce the cost of ownership of the network but comes with technical challenges, which this paper attempts to tackle.

An energy-harvesting IoT node harvests energy from its environment to recharge an energy-storage device and/or to directly power its components. In order to increase the amount of harvested energy, recent designs have introduced the possibility to simultaneously harvest energy from different sources [4,5]. More harvested energy allows the node to be powered for a longer time or to increase its QoS (Quality of Service). In the context of this work, the QoS of a node is defined as the quantity of data sensed and transmitted. When the node executes multiple tasks, the definition of QoS also includes the execution of multiple sensing and transmission tasks.

However, most energy-harvesting sources are variable and do not provide a constant power supply. Even if low-power IoT nodes are used, there is a risk of fully depleting the energy storage while no energy is harvested. In order to ensure continuous operation of the IoT node, the use of a power management system has been introduced in [6]. In such a system, the IoT node adapts its QoS to its energy capabilities, thus avoiding depleting its storage when energy can seldom be harvested and increasing its QoS when energy is plentiful.

The purpose of this work is to use technologies from both the energy-harvesting and energy-management fields and to design a production-ready autonomous IoT node based on an existing industrial platform. A multi-source and multi-task energy-harvesting LoRaWAN IoT node is presented and uses a power-management system to maximize its QoS and to ensure continuous operation. This IoT node is connected to a real commercial LoRaWAN standard network, including a network server and an application server for data presentation. The IoT node operates as a standard class C LoRaWAN device and is seen by the network as any other class C LoRaWAN device. Though the whole system is presented here, its different components are largely independent and could be reused with different hardware or software. Specifically, the contributions presented in this paper are as follows:

1. A multi-source energy-harvesting circuit based on off-the-shelf components that can harvest energy from a large variety of energy sources without a design change. This includes sources such as a solar panel, a low-voltage source such as a TEG (Thermo-Electric Generator), and any alternating voltage source such as a wind turbine or a piezoelectric generator. This platform is presented in Section 3.
2. An energy-allocation policy used to fairly allocate the harvested energy to multiple heterogeneous tasks. This design is explained in Section 4.2, from the theoretical optimal energy allocation calculation to the adaptation of these results to real-world conditions.
3. An implementation based on a real-world device and LoRaWAN network instead of simulation, which corresponds to an industrial use-case. This bridges the academic results and industrial constraints. The benefits of multi-source energy harvesting are measured and demonstrated, especially when complementary energy sources are used.

The rest of this paper is organized as follows. The state-of-the-art of different hardware platforms and power-management systems for IoT nodes is presented in Section 2. The hardware and software architectures of our LoRaWAN energy-harvesting IoT platform are then presented in Section 3. Section 4 details the designed energy-allocation systems for both single-task and multi-task IoT nodes. The experimental results obtained with the previously described energy-allocation policies are shown in Section 5. Finally, the conclusions and perspectives for future works are presented in Section 6.

## 2. Review of the Literature

In order to fulfill the requirements of all IoT applications, several wireless transmission protocols have been developed. LoRaWAN [1], based on LoRa [2] communications, is particularly suitable for remote data sensing, as it enables long-range communications with a reduced power consumption. LoRaWAN IoT nodes can therefore be deployed in remote areas with a small number of gateways, where energy harvesting is a relevant candidate as the power [7,8]. Such sensor networks can typically be deployed to increase the precision of phenomenon detection in remote areas [9,10,11].

Multiple energy-harvesting systems have been designed for different energy sources, such as solar panels [12], wind turbines [13], TEGs [14], or piezoelectric generators [15]. Additionally, different multi-source energy-harvesting systems have been designed to simultaneously harvest energy from different sources. In [16], a diode ORing system was used to connect multiple energy sources to a single power converter. However, this system does not use a MPPT (Maximum Power Point Tracking) [17,18] system for each source, which prevents the sources from delivering their maximum power. In [5], each source was alternatively connected to the power converter and stored its energy in a capacitor while it was disconnected. This system is efficient when sources have a similar voltage but requires more than three sources to be cost efficient. Ambimax [4] used a power converter and an energy storage for each source, which is more expensive but enables the use of an MPPT circuit per source. In our work, a similar architecture is used, but all of power converters are directly connected to a single energy storage through an integrated battery-management circuit. Another approach [19] is the use of a specific material able to harvest energy from solar, thermal, and kinetic sources. The use of such materials could remove the need for specific circuitry when energy is harvested from different sources.

In order to efficiently use the harvested energy, multiple techniques are used to reduce the component power consumption. Dynamic Frequency and Voltage Scaling (DVFS) [20] reduces the dynamic power consumption by lowering the operating voltage and frequency of the active components. Another method is to use duty cycling [21], where components are powered off or set in sleep mode while they are inactive. This method is especially efficient for IoT nodes, where the components are inactive most of the time as the device does not continuously measure and transmit data. However, even with a reduced energy consumption, an energy-harvesting IoT node can empty its energy storage in cases of energy scarcity. Thus, the node needs to integrate a power-management system to dynamically adapt its QoS to its energy capacities.

This type of power-management system was first proposed in [22], which uses an Exponentially Weighted Moving Average (EWMA) algorithm to predict the future harvested energy from a solar panel and accordingly adapts the node duty cycle. This system is extended in [23], which presents the Weather Conditioned Moving Average (WCMA), improving the prediction precision of EWMA by taking into account past and present weather measurements. These power managers are called “model-based”, as they expect the energy source to match a model of the source in order to predict its energy output. However, they are difficult to apply for unpredictable energy sources or for multi-source energy harvesting systems, where multiple energy sources are combined.

An alternative is to use “model-free” algorithms, which only take as data input current and past energy capabilities, such as the residual energy of the storage device $E_{R}$ and the harvested energy $E_{H}$. These algorithms are often close to control systems, in which a control loop dynamically adapts a controlled output value to match the input value variations. For instance, the authors of [24] used Proportional-Integral-Derivative (PID) control system to adapt the delay between two data transmissions based on the energy in a super-capacitor. LQ-tracker [25] uses a linear quadratic tracker that adapts the duty cycle to minimize the difference between the current residual energy and a target one, using only $E_{R}$ as the input. Fuzzyman [26] uses fuzzy logic to compute an energy budget, i.e., the quantity of harvested energy that can be used in a time slot, and use $E_{R}$ and $E_{H}$ as the inputs. RLman [27] is based on reinforcement learning but is limited to single-task IoT nodes.

Energy allocation for multi-task systems can be seen as a task scheduling problem, where tasks are constrained by both their energy consumption and QoS requirements instead of their deadline and/or period. Most energy-aware task scheduling policies [28,29,30,31,32,33] target real-time systems, for which the objective is to ensure that all tasks meet their deadline requirements instead of allocating energy to different tasks. DEOS [34] takes a different approach by considering energy as a schedulable resource to dynamically schedule tasks depending on their energy consumption and the available energy. Furthermore, DEOS is able to enforce QoS rules using the minimum and maximum number of tasks executed. Our work differs from DEOS as a mathematical approach is used to compute the optimal number of executions for each task. Moreover, our work considers the full system design, from energy harvesting hardware to energy management, while DEOS focuses solely on the task scheduling problem.

Although the use of long-range radio networks is picking up in recent years, most academic work on energy-harvesting IoT networks focus on mesh network technologies, and only a few articles discuss the use of LoRaWAN Energy-Harvesting IoT (EH-IoT) nodes. A LoRaWAN IoT node used for safety applications was presented in [35]. The node implements a functional power management system but does not detail it. Solar panels and a TEG were both used in [36] to power a floating LoRaWAN IoT node, but no power management was implemented. In our work, a LoRaWAN IoT node platform with flexible multi-source energy-harvesting capabilities and power-management features is presented. Our approach is entirely based on off-the-shelf components to ease implementation. Moreover, the case of IoT nodes that execute different sensing tasks is also considered.

## 3. Multi-Source Multi-Task Node Architecture

**Figure 1:**
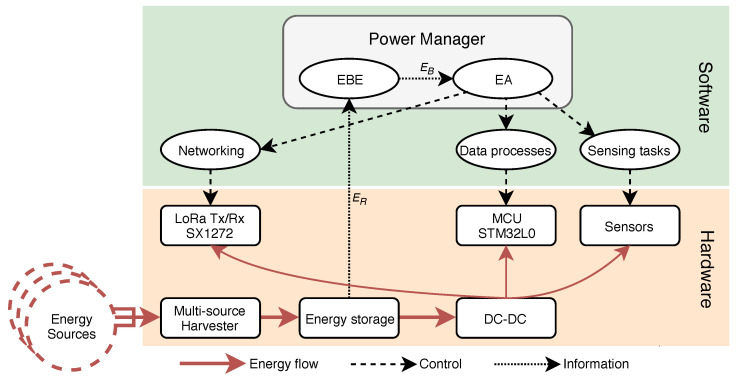
Full block diagram of the proposed energy harvesting IoT node.

In order to validate their design, the energy allocation policies proposed in this paper were implemented on a real multi-source EH-IoT node. The complete block diagram of the proposed node is shown in Figure 1. The hardware part is composed of two boards: a preexisting industrial LoRaWAN platform from Wi6labs and a multi-source energy-harvesting board. Both the networking protocol and power management software module are implemented on a microcontroller. The use of LoRaWAN in this work serves as a context, and no modifications or contributions are made to the LoRaWAN standard stack itself. The full platform is built using off-the-shelf components, making it easier to adopt for industrial applications.

The Wi6labs LoRaWAN platform is composed of a STM32 microcontroller, a SX1272 LoRa transceiver from Semtech, and their power supplies. The platform provides standard interfaces (I2C, SPI, and UART), GPIOs, and ADC inputs to connect multiple sensor boards depending on the target application. In this study, the sensor board includes a CO${}_{2}$ sensor, a noise level measurement system and a temperature/humidity sensor.

### 3.1. Multi-Source Energy-Harvesting Architecture

**Figure 2:**
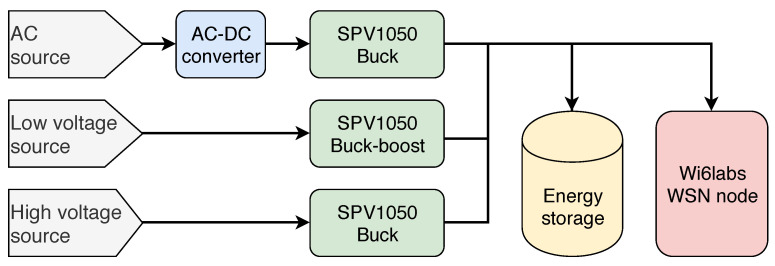
Architecture of the proposed multi-source energy-harvesting board.

The LoRaWAN platform is powered by a multi-source energy-harvesting board, described in Figure 2, which can combine up to three energy sources: a very low-voltage source, such as a TEG; an alternating voltage source, such as a wind turbine; and a voltage source up to 18 V, such as a solar panel. The board can therefore be used with most of the energy-harvesting sources used for IoT nodes. Moreover, it is possible to change the configuration of each input by changing the value of a few resistors, allowing the adaptation of the board to different applications without designing a new one. In order to ease its potential industrialization, the board is only composed of off-the-shelf components. The architectures allowing the simultaneous harvesting of multiple-energy sources have been previously explored in [5], and the results show that the architecture of Figure 2 gives the best performance. Although the architecture is straightforward, its implementation as well as its integration with energy management techniques in an industrial product are new.

Each input is connected to a SPV1050 [37] from STmicroelectronics, as shown in Figure 2. This component integrates a power converter, which can be configured as a boost or buck-boost regulator, a battery charger, and a MPPT circuit in order to maximize the harvested energy. The MPPT circuit used in this component is based on the Fractional Open-Circuit Voltage approach [38,39], which enables a small and cheap implementation and is precise enough for the targeted energy levels. To set its operating point at the correct voltage, the DC–DC converter varies its switching frequency. This modulates its input impedance and, thus, the voltage-operating point of the integrated circuit (IC) according to the MPPT circuit. Both the MPPT and battery charger thresholds can be configured with external resistors.

The SPV1050 stores the harvested energy in a capacitor $C_{STORE}$. This capacitor is connected to the battery when its voltage $V_{STORE}$ reaches the end-of-charge voltage $V_{EOC}$ and is disconnected when $V_{STORE}$ decreases under the under-voltage protection threshold $V_{UVP}$. Moreover, to avoid overcharging the battery, the integrated DC–DC converter is stopped when $V_{STORE}$ reaches $V_{EOC}$ until $V_{STORE}$ decreases under $V_{EOC}-EOC_{HYST}$, where $EOC_{HYST}$ is an hysteresis voltage set to 1% of $V_{EOC}$.

The use of this battery charging circuit ensures that the energy storage is neither charged nor discharged outside of the energy-storage specifications, i.e., $V_{STORE}$ stays bounded between $V_{UVP}$ and $V_{EOC}$. Thus, it is possible to directly connect each SPV1050 to a single energy storage. If one energy source does not provide enough power to charge the common energy storage but its capacitor $C_{STORE}$ is still connected to the energy storage (i.e. $V_{STORE}\geq V_{UVP}$), the SPV1050 is powered from the energy storage. A diode could prevent this current return, but the current consumption of the component (≤3 μA) is much lower than the return current of a diode. In the EH-IoT considered in this paper, the three SPV1050 are connected to a 7.5 F supercapacitor, which directly powers the IoT node. This supercapacitor has a maximum leakage current of 70 μA. This current is much lower than the ones provided by the energy sources used in our experimentation and, thus, is ignored to simplify the energy management.

### 3.2. Software Architecture

The considered EH-IoT node firmware is based on Contiki RTOS [40]. Besides the typical sensing, data processing, and LoRa transmission tasks, a power management module is also embedded to adapt the node behavior to its energy capabilities. As shown in [26], a power management system can be divided in two sub-blocks: an Energy Budget Estimator (EBE), which computes an energy budget $E_{B}$, which represents how much energy can be spent over a time slot, and an EA (Energy Allocator), which decides how the energy budget $E_{B}$ should be used. For multi-task systems, there is indeed an interest in separating how much energy can be used, which takes only energy capabilities into account, and how it is used, which should only take a task set and QoS requirements as inputs. As most previously designed power managers only take single-task devices into account, the energy-allocation step is implicit and the power manager can easily skip this step and deliver an $E_{B}$.

In state-of-the-art applications, the power management algorithm is executed at the end of fixed-duration time slots, and the computed duty cycle is applied for all transmissions during the next time slot. The duration of the time slot must be long enough to cover multiple transmissions. For example, many works use a time slot duration of one hour with mesh network communications, where the delay $D_{TX}$ between two transmissions varies from seconds to minutes. However, in the case of LoRaWAN communications, $D_{TX}$ can range from minutes to hours or even days, depending on the use case. This requires the use of very long time slots, and prevents the power management algorithm from converging to an optimized duty cycle in a reasonable time. To prevent this problem, the considered power manager is executed after each data transmission, so that the node can quickly adapt its behavior to variations in the environment.

The use of multi-source energy harvesting impacts the choice of EBE algorithms. As multiple energy sources can be used with the platform, no single model can be applied to estimate future harvested energy. Thus, it is not possible to use model-based EBE algorithms. Moreover, as the delay between two EBE executions can be long, it is necessary to use a model-free EBE algorithm that can converge towards an optimized duty cycle in a few executions. However, previous work [41] has shown that EBE algorithms have low performance differences when they are properly optimized, which enables the use of simple algorithms to compute $E_{B}$. For this study, LQ-tracker [25] is used as the EBE algorithm, since it provides good performance without requiring its parameters to be tuned.

The calculated $E_{B}$ then has to be allocated to one or more tasks. This is the role of the energy-allocation system. The choice of this system is mainly dependent on the applicative use case of the EH-IoT node. A distinction can be made between single-task and multi-task systems. In the first case, the EH-IoT has to execute a single task, which can be composed of multiple sub-tasks. An EH-IoT node that senses, processes, and transmits the data falls into this category. On the other hand, multi-task systems have to execute multiple tasks such as different types of measurement. In this case, the EA system aims to allocate the energy-budget $E_{B}$ according to their defined characteristics. These can include their priority, energy consumption, or QoS requirements set by the designer. This energy allocation subsystem is described in detail in Section 4. In this paper, both cases are considered and studied.

## 4. Energy Allocation for IoT Nodes

Energy allocation is the process of allocating an $E_{B}$ to one or more tasks. It can be noticed that, in some industrial use cases, the QoS can be constrained by a minimum and/or maximum number of transmissions and task executions. Therefore, the EA decides how many times a task is executed between two consecutive LoRa transmissions, separated by a delay $D_{TX}$ while taking into account these applicative constraints.

### 4.1. Single-Task Energy Allocation

This part first addresses the case of IoT nodes that only perform one task. Although this task can be composed of multiple sub-tasks, it is assumed that the task is executed as a whole. An example of such a system is a node that measures a value, processes it, and immediately transmits it over the network. This approach is typically used for measurement reporting applications. The energy consumption of this single task is supposed to be constant and known a priori, and is denoted $E_{C}^{mono}$. All the notations of this section are given in Table 1.

In terms of energy allocation, this approach is a relatively simple one. Indeed, in this use case, the goal of energy allocation is to convert the energy budget $E_{B}$ into an inversely proportional delay between two messages $D_{TX}$. The energy allocator computes a decreasing function $D_{TX}=f\left(E_{B}\right)$. This function can be designed so that its shape fits the application, e.g., it takes more risks by computing a smaller $D_{TX}$ for a large range of $E_{B}$ values, or is more conservative and delivers $D_{TX}=D_{TX}^{max}$ for a range of small $E_{B}$ values. Different functions can be used as long as it is a decreasing one. The allocation is either conservative or reactive depending on the choice of function.

The QoS is set in the library by defining a time reference D, and the minimal and maximal delays between two transmissions $D_{TX}^{min}$ and $D_{TX}^{max}$. The value of D is used to compute sleep duration values. A high D value enables longer low-power periods but reduces the granularity and precision of short ones. The minimal and maximal energy budget $E_{B}^{min}$ and $E_{B}^{max}$ for this application are computed as (1) and (2), respectively. In our case, D is set to one hour.

(1) $$E_{B}^{min}=\frac{D\times E_{C}^{mono}}{D_{TX}^{max}}$$

(2) $$E_{B}^{max}=\frac{D\times E_{C}^{mono}}{D_{TX}^{min}}$$

In this work, two functions for single-task energy allocation are introduced and evaluated. Both deliver a delay $D_{TX}$ between the minimal and maximal values $D_{TX}^{min}$ and $D_{TX}^{max}$, which are set as parameters by the system designer. The first function computes $D_{TX}$ between $D_{TX}^{min}$ and $D_{TX}^{max}$ as a pro-rata of $E_{B}$ between $E_{B}^{min}$ and $E_{B}^{max}$. Thus, the ramp function $D_{TX}=f\left(E_{B}\right)$ is given by the following:

(3) $$D_{TX}=D_{TX}^{max}-\frac{E_{B}-E_{B}^{min}}{E_{B}^{max}-E_{B}^{min}}\times \left(D_{TX}^{max}-D_{TX}^{min}\right).$$

The second function generalizes (1) and (2), which compute, respectively, $E_{B}^{min}$ and $E_{B}^{max}$ as functions of $E_{C}^{mono}$, D, $D_{TX}^{min}$, and $D_{TX}^{max}$:

(4) $$E_{B}=\frac{D\times E_{C}^{mono}}{D_{TX}}.$$

The delay $D_{TX}$ can then be extracted by the *inverse* function:

(5) $$D_{TX}=\frac{D\times E_{C}^{mono}}{E_{B}}.$$

Equation (5) is equivalent to a previous result obtained through a different reasoning [42] and by considering the power consumption in sleep mode negligible. This approximation holds in the case of long-range transmission as $E_{C}^{mono}$ is generally higher than in traditional mesh-networked IoT nodes. Indeed, in our use case, the delay between two task executions is constrained between 15 min and 3 h. Thus, the energy consumed in sleep mode (≤0.8 mJ, depending of duty cycle) is negligible compared to the energy burst consumed during a task execution (≈140 mJ) and can be considered equal to 0. The practical impact of this decision is that the computed $D_{TX}$ is slightly lower, thus increasing the QoS but slightly increasing the risk of energy storage depletion.

### 4.2. Multi-Task Energy Allocation

The multi-task case is significantly more complex than the single-task one. Several multi-task use cases can be considered, e.g., a single sensor that separates the sensing, processing, and transmission tasks or nodes that measure multiple physical values with different QoS requirements for each sensing task. An example of such a use case is the Super Citizen Smart Sensor (SCSS) project [43], in which nodes are able to measure noise level and gas concentration for air quality, temperature, and humidity. Each sensing task requires a different periodicity: temperature and humidity tend to change slowly, while the noise level can vary very quickly. Moreover, the power consumption of each task is different. An efficient energy allocation algorithm is therefore required.

The strategy for data transmission can also differ according to the use case. Measured data can be either directly transmitted after its measurement or aggregated with other measured data. The first strategy reduces the latency (i.e., the delay between a measurement and its transmission) to a minimum. However, it consumes more energy, as a larger number of transmissions is attempted. Thus, the QoS for all tasks is also reduced. If all tasks must be executed only once per transmission slot, the system can wake up, execute all sensing and processing tasks, transmit the relevant data, and go back into sleep mode. In this case, the system is analogous to a mono-task system, in which a meta task composed of all sensing/processing tasks and the transmission task is periodically executed. Finally, the transmission task can send aggregated data from multiple sensing and processing tasks. This last case is explored in this part, with a target of QoS maximization for all sensing tasks. The task set is considered constant, and the use of dynamic tasks is not considered in this work.

All the notations of this section are given in Table 2. In this work, a task $\tau_{i}$ is defined by the process it executes, its energy consumption $E_{C}^{\tau_{i}}$, its priority $\rho_{i}\in \left[0;1\right]$, and its number of executions between two transmissions $\phi_{i}$. $\phi_{i}$ has minimal and maximal values, denoted by $\phi_{i}^{min}$ and $\phi_{i}^{max}$, respectively, which enable us to set the required QoS of the task. A high-priority task has a high $\rho_{i}$. A task for which execution is not always required can have $\phi_{i}^{min}$ set to 0. The transmission task is a particular one, as it is always executed at the end of a time slot and can be modeled by setting both $\phi_{i}^{min}$ and $\phi_{i}^{max}$ to 1. The energy consumption of the transmission task is denoted $E_{C}^{TX}$. This task model only adds QoS requirements and energy awareness to processes. Real-time capabilities can be integrated either by linking energy-aware tasks to real-time processes, which embed information about their deadline and/or period, or by adding this information to the task model.

For multi-task systems, $E_{B}^{min}$ corresponds to the case where all tasks are executed the minimal number of times $\phi_{i}^{min}$ with a delay $D_{TX}=D_{TX}^{max}$ between two transmissions and thus can be expressed by the following:

(6) $$E_{B}^{min}=\frac{D\times \sum_{i=1}^{K}\left(\phi_{i}^{min}\times E_{C}^{\tau_{i}}\right)}{D_{TX}^{max}},$$

with *K* being the total number of tasks.

Inversely, $E_{B}^{max}$ corresponds to the case where each task $\tau_{i}$ is executed $\phi_{i}^{max}$ times with a delay $D_{TX}^{min}$ between two transmissions:

(7) $$E_{B}^{max}=\frac{D\times \sum_{i=1}^{K}\left(\phi_{i}^{max}\times E_{C}^{\tau_{i}}\right)}{D_{TX}^{min}}.$$

The goal of the EA is the maximization of the QoS for all sensing tasks, i.e., maximizing $\phi_{i},\;\forall i\in \left\{1,\cdots ,K\right\}$. The energy-allocation problem can be formulated by (8) and its goal is to maximize the consumed energy $E_{C}^{Total}$, constrained by the energy budget $E_{C}^{Total}\leq E_{B}-E_{C}^{TX}$.

(8) $$\begin{array}{c} \max_{\phi_{i}}\left(E_{C}^{Total}=\sum_{i=1}^{K}\left(\phi_{i}\times E_{C}^{\tau_{i}}\right)\right)\\ s.t.\;\;E_{C}^{Total}\leq E_{B}-E_{C}^{TX}\;\;\;\;\;\;\;\;\;\; \end{array}$$

To solve this problem, a first approach is to use purely algorithmic solutions to find the set $\left\{\phi_{1},\phi_{2},\cdots ,\phi_{K}\right\}$ that maximizes $E_{C}^{Total}$ while still keeping $E_{C}^{Total}\leq E_{B}-E_{C}^{TX}$. Such an algorithm has to compute all possible combinations of $\phi_{i}$ and to select the one that best respects both energy budget and QoS constraints and the task priorities. However, its complexity is growing quickly with the number of tasks and requires both memory and computing resources that are not always available in an IoT node. As the solution space is very important, a fully algorithmic energy allocator will provide a suboptimal solution. There is therefore a need to determine an optimal solution to this problem. To solve this problem, we suppose the following hypotheses:

- All priorities $\rho_{i}$ are normalized so that $\sum_{i=1}^{K}\rho_{i}=1$
- Data are transmitted only once, at the end of each time slot.

This second assumption stems from the use of LoRaWAN as the transmission network. Many state-of-the-art energy management algorithms use mesh-networking technologies to transmit data, for which the delay between two messages ranges from seconds to minutes. In such a case, a time slot has a fixed duration that covers multiple transmissions. In our use case, the delay between two LoRaWAN messages can range from minutes to hours or even days in extreme cases. A fixed duration time slot long enough to cover multiple transmissions would lead to a long convergence time of the energy management algorithms and would decrease their stability. Thus, a variable duration time slot is chosen, defined as the time between two transmissions.

The allocation problem solution must be able to provide equity between the different tasks to avoid a task with a much lower consumption $E_{C}^{\tau_{i}}$ or much higher priority $\rho_{i}$ being the only one executed. One way to solve this problem while keeping fairness between tasks is to solve the cost function as a sum of logarithms [44], as defined in (9), where $x=\left[\phi_{1},\phi_{2},\cdots ,\phi_{K}\right]$ is a solution vector:

(9) $$f_{0}\left(x\right)=-\sum_{i=1}^{K}\left(\rho_{i}\times \ln\left(\phi_{i}\right)\right),$$

with the constraint defined by the function $g_{i}\left(x\right)$:

(10) $$g_{i}\left(x\right)=\left[\sum_{i}^{K}\left(\phi_{i}\times E_{C}^{\tau_{i}}\right)\right]-\left(E_{B}-E_{C}^{TX}\right)\leq 0.$$

This is a linear optimization of a convex function problem; thus, a local solution is also a global solution. The Lagrangian function for the problem can be expressed as follows:

(11) $$L\left(x,\lambda \right)=f_{0}\left(x\right)+\sum_{i=1}^{K}\left(\lambda_{i}\times g_{i}\left(x\right)\right),$$

with λ being a vector composed of the Lagrange multipliers $\lambda_{i}$. This optimization problem is solved using the KKT conditions [45]. This theorem states that, for each point x that maximizes the $f_{0}\left(x\right)$ function, there exists a set of these multipliers that satisfies the following conditions:

1. $g_{i}\left(x\right)\leq 0$
2. $\lambda_{i}\geq 0$, at least one λ is >0
3. $\lambda_{i}\times g_{i}\left(x\right)=0$

Thus, if a local solution satisfies the KKT conditions, this solution is proven to globally maximize the function and is the optimal allocated energy value. To determine this local solution, we pose that i=K (11) becomes the following:

(12) $$L\left(x,\lambda \right)=-\rho_{K}\times \ln\left(\phi_{K}\right)+\lambda_{K}\times \phi_{K}\times E_{C}^{\tau_{K}}-\left(E_{B}-E_{C}^{TX}\right).$$

This function of $\phi_{K}$ presents a local maximum when its derivative with respect to $\phi_{K}$ is equal to 0, expressed as follows:

(13) $$\begin{array}{c} \frac{dL\left(x,\lambda \right)}{d\phi_{K}}=-\frac{\rho_{K}}{\phi_{K}}+\lambda_{K}\times E_{C}^{\tau_{K}}=0\\ \Leftrightarrow \phi_{K}=\frac{\rho_{K}}{\lambda_{K}\times E_{C}^{\tau_{K}}}\;\;\;\;\;\;\;\;\;\;\;\; \end{array}$$

A local solution is achieved with $\lambda_{K}>0$ (as $\phi_{K}\geq 0$) and thus respects the first and second KKT conditions. If the third KKT condition is checked for $\lambda_{i}=\lambda_{K}\geq 0$, it becomes the following:

(14) $$\begin{array}{c} \lambda_{K}\times \left(\sum_{i=1}^{K}\left(\phi_{i}\times E_{C}^{\tau_{i}}\right)-\left(E_{B}-E_{C}^{TX}\right)\right)=0. \end{array}$$

$\lambda_{K}$ cannot be equal to 0; otherwise, $\phi_{K}$ would not exist. By integrating the local solution in (14), we obtain the following (15):

(15) $$\begin{array}{c} \sum_{i=1}^{K}\left(\frac{\rho_{i}}{\lambda_{K}\times E_{C}^{\tau_{i}}}\times E_{C}^{\tau_{i}}\right)=\left(E_{B}-E_{C}^{TX}\right)\\ \Leftrightarrow \frac{1}{\lambda_{K}}\sum_{i=1}^{K}\rho_{i}=\left(E_{B}-E_{C}^{TX}\right)\;\;\;\;\;\;\;\; \end{array}$$

As $\rho_{i}$ values are normalized so that their sum is equal to 1, (15) can be simplified as follows:

(16) $$\frac{1}{\lambda_{K}}=\left(E_{B}-E_{C}^{TX}\right)\Leftrightarrow \lambda_{K}=\frac{1}{E_{B}-E_{C}^{TX}}.$$

Thus, if $E_{B}>E_{C}^{TX}$, (16) proves that we have a set of $\lambda_{i}$ defined as $\lambda_{i}=\lambda_{K}=1/\left(E_{B}-E_{C}^{TX}\right)>0$, a solution that fulfills both the second and third conditions while the first condition is fulfilled by the fact that the function is convex. Therefore, the solution $\phi_{i}=\rho_{i}/\left(\lambda_{K}\times E_{C}^{\tau_{i}}\right)$ maximizes $f_{0}\left(x\right)$, provided that $E_{B}$ enables at least one transmission. If $E_{B}<E_{C}^{TX}$, the problem does not apply, as the IoT node does not have enough energy to perform any action.

Hence, the optimal $\phi_{i}$ for each task can be computed as follows:

(17) $$\phi_{i}=\frac{\rho_{i}\times \left(E_{B}-E_{C}^{TX}\right)}{E_{C}^{\tau_{i}}}.$$

Although this result is quite simple to compute, the mathematical process behind its demonstration ensures its optimality. It can be noticed that the result of this optimal formula is not always an integer value. It is possible to truncate each $\phi_{i}$ to obtain integer values that respect the constraint $E_{C}^{Total}\leq E_{B}-E_{C}^{TX}$. This enables computing the delay $D_{\tau_{i}}$ of each task and scheduling them as with the previous algorithmic method. However, that breaks the optimality, as there will still be a difference between $E_{C}^{Total}$ and $E_{B}-E_{C}^{TX}$.

Another possibility is to compute $D_{\tau_{i}}=D_{TX}/\phi_{i}$ while keeping the exact value of $\phi_{i}$, even if it is not an integer. As it becomes much complicated to compute a greatest common divisor of the different $\phi_{i}$, each task must be scheduled with its own timer. Although this increases the computing requirements, most embedded operating systems provide software timer libraries that simplify this implementation and do not require a large number of hardware timers. Each timer can be set to the new $D_{\tau_{i}}$ value when it is computed. As the tasks are run asynchronously from the transmission task and the time slot, the data to be transmitted must be stored in a properly sized buffer. In the case of long-range transmission, such as LoRaWAN networks, where only a few tens of bytes can be transmitted at once, the QoS of the tasks must be set carefully to avoid overflowing this transmission buffer and losing data.

Although this solution is optimal with regard to the priority $\rho_{i}$ and energy consumption $E_{C}^{\tau_{i}}$ of the tasks, it does not take into account the minimal and maximal QoS of the tasks. Moreover, it does not compute the delay between two consecutive transmissions, as it is supposed that each time slot ends with a transmission. Thus, these computations have to be included in an algorithm that enforces the required QoS and computes an adapted $D_{TX}$.

Algorithm 1 proposes the integration of the optimal $\phi_{i}$. It defines $E_{C}^{max}$ as the maximum energy that can be consumed, i.e., the $E_{C}^{Total}$ when all $\phi_{i}$ are equal to $\phi_{i}^{max}$ and $D_{TX}=D_{TX}^{min}$. If $E_{B}>E_{C}^{max}$, it enforces the maximal QoS rules, which lets the node save more energy for later usage. If $E_{B}<E_{C}^{max}$, it tries to maximize the QoS by allocating energy using the optimal formula in (17). This example favors the battery life over the QoS, as the minimal QoS settings are not enforced if not enough energy is available. On the other hand, it tries to maximize QoS as much as possible, ensuring that high-priority tasks are favored. It can be noticed that the QoS limits do not have to be aligned with the priority setting. For example, a high-priority task may require only one execution per timeslot while a less important sensing task may be executed multiple times. Due to this, the calculated $\phi_{i}$ must be capped at $\phi_{i}^{max}$. Although this breaks the optimality of the $\phi_{i}$ computation for the other tasks, it avoids spending energy on an unrequired task execution.
**Algorithm 1:** Optimal $\phi_{i}$ calculation integrated in a practical solution
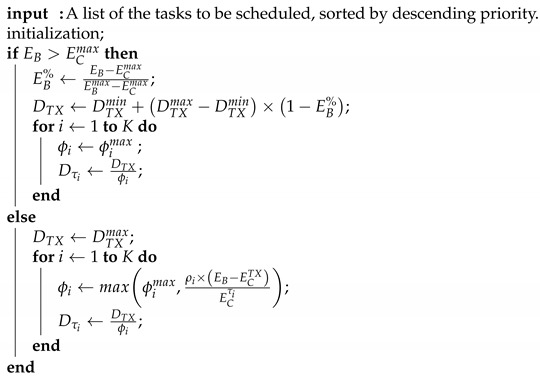


## 5. Experimental Validation

**Figure 3:**
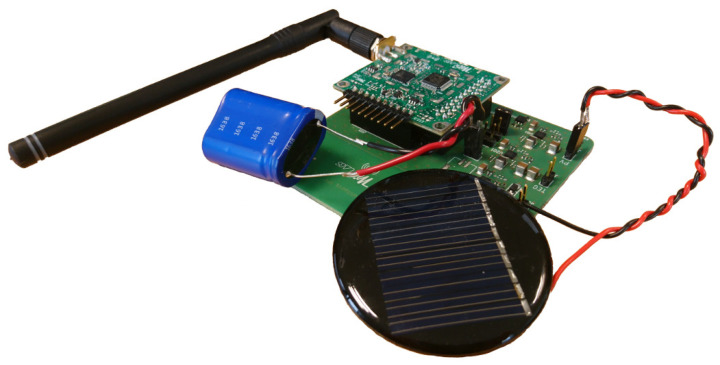
Single-task IoT node platform.

All proposed energy allocators were implemented, tested, and compared on a real LoRaWAN IoT node, shown in Figure 3. This implementation was performed in an external library, and neither the base OS nor the tasks needed to be modified. This node was based on the Wi6labs platform and the multi-source energy-harvesting board described in Section 3. LQ-tracker was used as the EBE algorithm. All functions were implemented as a portable C library to help validation and to speed up the development. During the experiments, all of the nodes were connected to a commercial IoT network and reported the measured data to a real application server. Thus, the experiments were executed and validated in a real-world scenario. These experiments also validated the approach of multi-source energy harvesting to increase the QoS of an IoT node.

### 5.1. Single-Task Energy-Allocation Results

Two nodes were deployed, each powered by a small solar panel with a maximum voltage of 5 V for 40 mA. The IoT node firmware consists of a single task, composed of a temperature and humidity measurement and its transmission over the LoRaWAN network. Both nodes used LQ-Tracker as the EBE algorithm. The first one used the ramp function (3) and the other used the inverse function (5). Both IoT sensor nodes were run at the same location for approximately 40 days, sending logs of each transmission and energy capabilities over an UART link to a computer. During the campaign, the energy-storage voltage $V_{BAT}$, the energy budget $E_{B}$, the delay between two transmissions $D_{TX}$, and the number of successful transmissions were recorded. The results of this experiment are shown in Table 3.

The use of the ramp function implements a more conservative policy than the inverse function. This induces a higher delay $D_{TX}$ despite a higher energy budget $E_{B}$, which enables the node to keep more energy in storage, as shown by the increase in the mean $V_{BAT}$ value, and to decrease the QoS as less messages are transmitted. This experiment shows that, for single-task IoT nodes, the choice of the $D_{TX}=f\left(E_{B}\right)$ EA function is a compromise between QoS and energy safety.

### 5.2. Multi-Task Energy-Allocation Results

Algorithm 1 was implemented and evaluated on real-world IoT nodes. The node firmware provides lightweight software timer libraries. Thus, an asynchronous task execution scheme, where all tasks have their own timer, can be implemented with a low overhead. The delay for each timer was computed using the proposed algorithm.

This multi-task energy allocation scheme was implemented on two sensor nodes with two different energy-harvesting sources. Each node was equipped with a temperature/humidity sensor, a CO_2_ sensor, and a noise sensor. The power consumption characteristics and minimal/maximal QoS settings for each sensor are described in Table 4. In this experiment, all priorities were set to the same value. It is clear that a higher priority leads to more task executions. Setting an equal value for all tasks priority avoids this effect and highlights the influence of QoS requirements on the energy allocation.

The delay between two LoRa transmissions is heavily dependent on the use case and should be adapted depending on the application. In our system, it was set to vary between 15 min to 3 h. A minimal delay $D_{TX}^{min}$ was required as LoRa transmissions operate in Industrial, Scientific, and Medical (ISM) bands, which are free to use but limit the transmission airtime of the device. Moreover, too frequent transmissions could drain the battery and make the system less stable. The maximum delay $D_{TX}^{max}$ was set to enforce a minimum QoS. In our use case, $D_{TX}^{max}$ was set to 3 h, as we considered it a reasonable minimum QoS for meteorological data, which tends to change slowly. These delays should be adapted to each application use case by the system designer.

**Figure 4:**
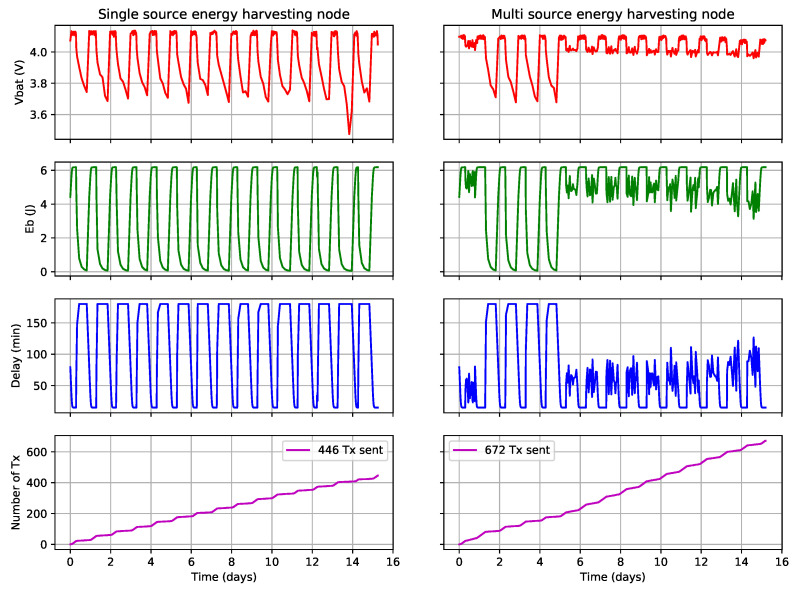
Experimental measurement of single and multi- source energy harvesting systems.

The first sensor was powered by a 2 W solar panel, while the second sensor used a multi-source energy-harvesting board to combine two solar panels capable of up to 2 W and 3.5 W. Additionally, a voltage generator was used to emulate a TEG with an open voltage of 800 mV and a short-circuit maximum current of 4 mA. Taking into account the MPPT circuit and power converter efficiency, this simulated source delivers a continuous power of 0.64 mW, which is small enough to let the 7.5 F supercapacitor partially discharge. In addition to the previous measurements, the number of failed transmissions was also recorded. These measurements are shown in Figure 4.

The experiment was run for a total of 15 days. The emulated TEG was turned off during the second day in order to test the efficiency of multi-source energy harvesting with similar types of energy sources, i.e., two solar panels. The emulated TEG was turned on at the end of the fifth day.

This experiment first shows that such a system is functional with real-world platforms and use cases. The solution is implemented as an add-on and does not modify the existing hardware, while the energy management software only extends the firmware without modifying the existing tasks or the underlying OS. The measurements show that, as expected, the use of multiple energy-harvesting sources has a positive impact on the QoS of the node. In particular, Table 5 shows that the mean $E_{B}$ is significantly higher and that the mean $D_{TX}$ is lower when more energy-harvesting sources are used. Due to this, the node with multiple sources is able to transmit 672 packets, compared to 446 packets when the node is powered by a single energy source. In both cases, no transmission failed due to a low $E_{R}$. These results show that multi-source energy harvesting is a valid way to increase the QoS of IoT nodes. Furthermore, the use of data aggregation enables the node to maximize the QoS by reducing the number of transmissions, thus saving power via radio sleep.

It can be noticed that the type of energy sources used has a direct impact on the efficiency of the multi-source energy-harvesting system. From the second to the fifth days, the first node was powered by two solar panels, providing, respectively, 2 and 3.5 W while the second node was only powered by a 2 W solar panel. During this period, the multi-source energy-harvesting node was only marginally more efficient than the single-source energy-harvesting node, with 141 transmitted packets against 133. Indeed, the 2 W solar panel is sufficient to completely recharge the energy storage and to maximize its QoS at the start of a new day. Thus, the additional solar panel only helps the node recharge its storage faster and additional energy is not required during the day.

On the contrary, when a TEG is used during day and night, its energy enables the node to significantly increase the QoS. In this case, the simulated TEG complements the solar panel, bringing energy when solar panels do not harvest energy. This shows that the use of a second similar source has little interest in some use cases while a complementary energy source would have a higher impact on the QoS of the node.

**Figure 5:**
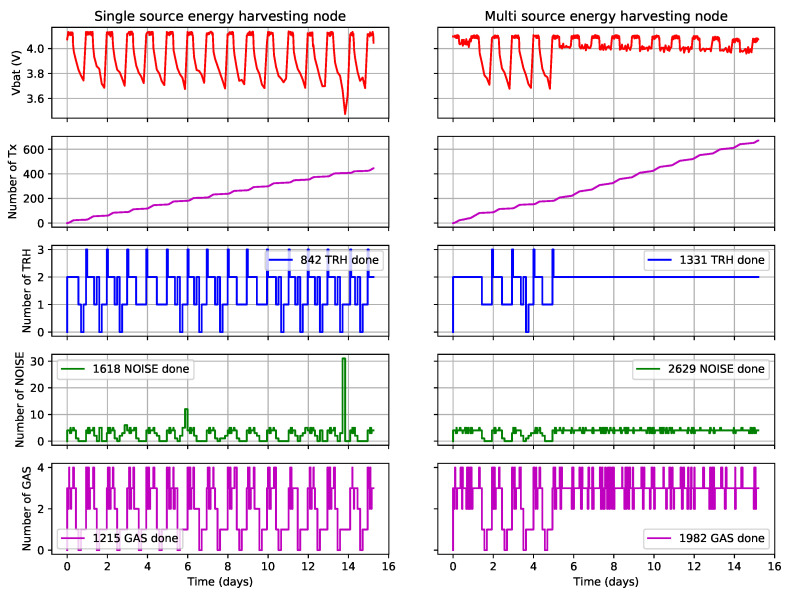
Number of task executions for single- and multi-source energy-harvesting systems.

Additionally, the number of executions per time slot is counted for each task. Thus, the mean and total number of executions can be computed for each task. These results are presented in Table 6, while Figure 5 shows the distribution of the measurements over time.

### 5.3. Discussion

Overall, the use of multi-source energy-harvesting increases the number of executed sensing tasks by 62% and the number of transmitted messages by 51%. This shows that our energy allocation system works as expected and enables the multi-source energy-harvesting node to execute significantly more tasks than the single source one. These results confirm that the addition of a second solar panel has much less effect on the node QoS than the use of the emulated TEG. Indeed, the emulated TEG is complementary to the solar panels, as it provides power at night when the solar panels does not. This can be seen in the distribution of task execution, which is similar between the two nodes when the emulated TEG is powered off. When it is powered on, however, the multi-source energy-harvesting node has enough energy to maximize the QoS of each task and to reduce the delay $D_{TX}$ between transmissions while the other node still has to modulate the number of executions of each task to avoid depleting its battery. This demonstrates that the energy allocation works as expected: the multi-source energy-harvesting node executes significantly more tasks than the single-source one.

In some time slots, the number of executions of some tasks is higher than the specified maximum. This is due to the fact that each task manages its own timer. In some situations where multiple timers expire at a similar time, a task execution is already scheduled before its new number of executions is calculated. As the scheduler does not check if the task is already in the execution queue, it does not check this task execution, which can lead to an additional task execution. A bug also occurred around days 6 and 14, when the single-source energy-harvesting node, respectively, executed 12 and 31 noise sensing tasks, far more than specified in Table 4. However, these bugs are linked to implementation and do not prevent proper application of the energy-allocation policy.

In this case, the use of additional energy sources enable the multi-source energy-harvesting node to keep executing its tasks at their maximum QoS. Due to this, the number of task executions per time slot is much more stable, as seen with the lower standard deviation of this value in Table 6. Thus, the use of additional energy sources enables both a higher and more predictable QoS.

## 6. Conclusions

In this paper, a multi-task LoRaWAN IoT node with multi-source energy harvesting is presented and the associated power management system is detailed. We showed that an energy budget can be optimally allocated to multiple sensing tasks using simple calculations. This method was implemented and demonstrated on a real industrial hardware platform connected to a commercial LoRaWAN network. The presented system highlights how the power manager can be used to efficiently allocated an energy budget to heterogeneous tasks. Furthermore, the use of a multi-source energy-harvesting system enabled the same IoT node, in the same conditions, to execute 62% more sensing tasks and to transmit 51% more LoRaWAN messages. This demonstrates the interest in multi-source energy harvesting to increase the QoS of IoT nodes, especially when complementary energy sources are used. This also shows that current research results can easily be integrated into existing IoT nodes, which should help system designers reduce the reliance on single-use batteries in the future. This paper is significant as it associates the fields of energy harvesting and power management to present a whole system. Moreover, it bridges academic excellence and industry by applying state-of-the-art research results to an existing production-ready platform. Thus, it shows how existing platforms and products can be adapted to use energy harvesting with no costly architecture change.

## Figures and Tables

**Table 1:** Table of Notation—Section 4.1.

$E_{B}$	Energy budget
$E_{B}^{max}$	Maximal energy budget
$E_{B}^{min}$	Minimal energy budget
$E_{C}^{mono}$	Energy consumption of a task
$D_{TX}$	Delay between two consecutive transmissions
$D_{TX}^{max}$	Maximal delay between two consecutive transmissions
$D_{TX}^{min}$	Minimal delay between two consecutive transmissions
D	Time reference

**Table 2:** Table of Notation—Section 4.2.

$\tau_{i}$	Task *i*
$\rho_{i}$	Priority of task *i*
$E_{C}^{\tau_{i}}$	Energy consumption of task *i*
$\phi_{i}$	Number of executions of task *i* in a time slot
$\phi_{i}^{max}$	Maximum number of executions of task *i* in a time slot
$\phi_{i}^{min}$	Minimum number of executions of task *i* in a time slot
$E_{C}^{TX}$	Energy consumption of the transmission task
$E_{C}^{Total}$	Energy consumption of the task set
$E_{B}$	Energy budget
$E_{B}^{max}$	Maximal energy budget
$E_{B}^{min}$	Minimal energy budget
D	Time reference
$D_{TX}$	Delay between two consecutive transmissions
$D_{TX}^{max}$	Maximal delay between two consecutive transmissions
$D_{TX}^{min}$	Minimal delay between two consecutive transmissions
$D_{\tau_{i}}$	Delay between two task *i* executions

**Table 3:** EA algorithm comparison over 40 days.

EA		Inverse	Ramp
$V_{BAT}$(V)	Mean	3.962	4.002
	Std. deviation	0.075	0.064
$E_{B}$(J)	Mean	0.477	0.527
	Std. deviation	0.136	0.084
$D_{TX}$(min)	Mean	22.3	25.5
	Std. deviation	21.6	26.7
Transmitted messages		2362	2130

**Table 4:** Characteristics of the tasks executed by the multi-task energy harvesting nodes.

Sensing Task	Energy Consumption (J)	Minimal Number of Executions	Maximal Number of Execution
Temperature/Humidity	0.098	1	2
Noise	0.209	0	4
Gas (CO_2_)	0.172	1	3

**Table 5:** Performance of single- and multi-source energy-harvesting systems.

Energy-Harvesting Node		Single Source	Multi-Sources
$V_{BAT}$ (V)	Mean	4.058	4.061
	Std. deviation	0.061	0.127
$E_{B}$ (J)	Mean	5.064	5.682
	Std. deviation	1.095	2.123
$D_{TX}$ (min)	Mean	48.38	31.99
	Std. deviation	61.08	34.32
Messages transmitted		446	672
Failed transmission		0	0

**Table 6:** Energy allocator performance for single- and multi-source energy harvesting systems.

Task Executions		Single Source	Multi-Sources
Temperature/Humidity	Mean per time slot	1.871	1.978
	Std. deviation	0.482	0.199
	Total number of executions	842	1331
Noise	Mean per time slot	3.596	3.906
	Std. deviation	0.714	1.894
	Total number of executions	1618	2629
Gas	Mean per time slot	2.700	2.945
	Std. deviation	0.570	0.912
	Total number of executions	1215	1982

## Data Availability

Not applicable.

## References

[1] Sornin N., Luis M., Eirich T., Kramp T., Hersent O. (2015). LoRaWAN? Specification.

[2] Semtech Semtech LoRa Technology Overview. https://www.semtech.com/lora.

[3] Gartner Inc. (2017). Gartner Says 8.4 Billion Connected “Things” Will Be in Use in 2017, Up 31 Percent From 2016. https://www.information-age.com/gartner-8-4-billion-iot-2017-123464337/.

[4] Park C., Chou P. AmbiMax: Autonomous Energy Harvesting Platform for Multi-Supply Wireless Sensor Nodes. Proceedings of the IEEE Communications Society on Sensor and Ad Hoc Communications and Networks.

[5] Gleonec P.D., Ardouin J., Gautier M., Berder O. Architecture exploration of multi-source energy harvester for IoT nodes. Proceedings of the IEEE Online Conference on Green Communications (OnlineGreenComm).

[6] Kansal A., Hsu J., Zahedi S., Srivastava M.B. (2007). Power Management in Energy Harvesting Sensor Networks. ACM Trans. Embed. Comput. Syst..

[7] Mabon M., Gautier M., Vrigneau B., Le Gentil M., Berder O. (2019). The Smaller the Better: Designing Solar Energy Harvesting Sensor Nodes for Long-Range Monitoring. Wirel. Commun. Mob. Comput..

[8] Magno M., Ait Aoudia F., Gautier M., Berder O., Benini L. WULoRa: An Energy Efficient IoT End-Node for Energy Harvesting and Heterogeneous Communication. Proceedings of the IEEE/ACM Design, Automation & Test in Europe Conference & Exhibition (DATE).

[9] Tarighati A., Gross J., Jaldén J. (2017). Decentralized Hypothesis Testing in Energy Harvesting Wireless Sensor Networks. IEEE Trans. Signal Process..

[10] Ciuonzo D., Gelli G., Pescapé A., Verde F. Decision Fusion Rules in Ambient Backscatter Wireless Sensor Networks. Proceedings of the 2019 IEEE 30th Annual International Symposium on Personal, Indoor and Mobile Radio Communications (PIMRC).

[11] Ciuonzo D., Rossi P.S. (2018). Quantizer Design for Generalized Locally Optimum Detectors in Wireless Sensor Networks. IEEE Wirel. Commun. Lett..

[12] Raghunathan V., Kansal A., Hsu J., Friedman J., Srivastava M. Design considerations for solar energy harvesting wireless embedded systems. Proceedings of the IEEE International Symposium on Information Processing in Sensor Networks (ISPN).

[13] Porcarelli D., Spenza D., Brunelli D., Cammarano A., Petrioli C., Benini L. (2015). Adaptive Rectifier Driven by Power Intake Predictors for Wind Energy Harvesting Sensor Networks. IEEE J. Emerg. Sel. Top. Power Electron..

[14] Rossi M., Rizzon L., Fait M., Passerone R., Brunelli D. (2014). Energy Neutral Wireless Sensing for Server Farms Monitoring. IEEE J. Emerg. Sel. Top. Circuits Syst..

[15] Li W., Siyuan H., Shudong Y. (2010). Improving Power Density of a Cantilever Piezoelectric Power Harvester Through a Curved L-Shaped Proof Mass. IEEE Trans. Ind. Electron..

[16] Tan Y.K., Panda S.K. (2011). Energy Harvesting From Hybrid Indoor Ambient Light and Thermal Energy Sources for Enhanced Performance of Wireless Sensor Nodes. IEEE Trans. Ind. Electron..

[17] Kim H., Min Y., Jeong C., Kim K., Kim C., Kim S. (2013). A 1-mW Solar-Energy-Harvesting Circuit Using an Adaptive MPPT With a SAR and a Counter. IEEE Trans. Circuits Syst. Express Briefs.

[18] Lu C., Tsui C.Y., Ki W.H. (2011). Vibration Energy Scavenging System With Maximum Power Tracking for Micropower Applications. IEEE Trans. Very Large Scale Integr. Syst..

[19] Bai Y., Tofel P., Palosaari J., Jantunen H., Juuti J. (2017). A Game Changer: A Multifunctional Perovskite Exhibiting Giant Ferroelectricity and Narrow Bandgap with Potential Application in a Truly Monolithic Multienergy Harvester or Sensor. Adv. Mater..

[20] Snowdon D., Ruocco S., Heiser G. Power management and dynamic voltage scaling: Myths and facts. Proceedings of the ACM Workshop on Power Aware Real-Time Computing.

[21] Hsu J., Zahedi S., Kansal A., Srivastava M., Raghunathan V. Adaptive Duty Cycling for Energy Harvesting Systems. Proceedings of the IEEE International Symposium on Low Power Electronics and Design (ISLPED).

[22] Kansal A., Hsu J., Srivastava M., Raqhunathan V. Harvesting aware power management for sensor networks. Proceedings of the ACM/IEEE Design Automation Conference.

[23] Recas Piorno J., Bergonzini C., Atienza D., Simunic Rosing T. Prediction and management in energy harvested wireless sensor nodes. Proceedings of the IEEE International Conference on Wireless Communication, Vehicular Technology, Information Theory and Aerospace & Electronic Systems Technology.

[24] Le T.N., Sentieys O., Berder O., Pegatoquet A., Belleudy C. Power Manager with PID Controller in Energy Harvesting Wireless Sensor Networks. Proceedings of the IEEE International Conference on Green Computing and Communications.

[25] Vigorito C.M., Ganesan D., Barto A.G. Adaptive Control of Duty Cycling in Energy- Harvesting Wireless Sensor Networks. Proceedings of the IEEE Communications Society Conference on Sensor, Mesh and Ad Hoc Communications and Networks (SECON).

[26] Ait Aoudia F., Gautier M., Berder O. Fuzzy Power Management for Energy Harvesting Wireless Sensor Nodes. Proceedings of the IEEE International Conference on Communications (ICC).

[27] Ait Aoudia F., Gautier M., Berder O. Learning to survive: Achieving energy neutrality in wireless sensor networks using reinforcement learning. Proceedings of the IEEE International Conference on Communications (ICC).

[28] Moser C., Brunelli D., Thiele L., Benini L. Lazy Scheduling for Energy Harvesting Sensor Nodes. Proceedings of the IFIP Working Conference on Distributed and Parallel Embedded Systems (DIPES).

[29] Moser C., Brunelli D., Thiele L., Benini L. (2007). Real-time scheduling for energy harvesting sensor nodes. Real Time Syst..

[30] Chandarli Y., Abdeddaim Y., Masson D. The Fixed Priority Scheduling Problem for Energy Harvesting Real-Time Systems. Proceedings of the IEEE International Conference on Embedded and Real-Time Computing Systems and Applications.

[31] El Ghor H., Chetto M., Chehade R.H. (2011). A real-time scheduling framework for embedded systems with environmental energy harvesting. Elsevier J. Comput. Electr. Eng..

[32] Rao V.S., Prasad R.V., Niemegeers I.G.M.M. Optimal task scheduling policy in energy harvesting wireless sensor networks. Proceedings of the IEEE Wireless Communications and Networking Conference (WCNC).

[33] Audet D., MacMillan N., Marinakis D., Kui W. Scheduling recurring tasks in energy harvesting sensors. Proceedings of the IEEE Conference on Computer Communications Workshops (INFOCOM WKSHPS).

[34] Zhu T., Mohaisen A., Ping Y., Towsley D. DEOS: Dynamic energy-oriented scheduling for sustainable wireless sensor networks. Proceedings of the 2012 Proceedings IEEE INFOCOM.

[35] Wu F., Redouté J., Yuce M.R. (2018). WE-Safe: A Self-Powered Wearable IoT Sensor Network for Safety Applications Based on LoRa. IEEE Access.

[36] Lee W., Schubert M.J.W., Ooi B., Ho S.J. (2018). Multi-Source Energy Harvesting and Storage for Floating Wireless Sensor Network Nodes With Long Range Communication Capability. IEEE Trans. Ind. Appl..

[37] STMicroelectronics SPV1050—Ultra Low Power Energy Harvester and Battery Charger with Embedded MPPT and LDOs. https://www.st.com/en/power-management/spv1050.html.

[38] Ahmad J. A fractional open circuit voltage based maximum power point tracker for photovoltaic arrays. Proceedings of the 2010 2nd International Conference on Software Technology and Engineering.

[39] Ahmad J., Kim H.J. (2009). A Voltage Based Maximum Power Point Tracker for Low Power and Low Cost Photovoltaic Applications. WASET Int. J. Electron. Commun. Eng..

[40] Contiki Contiki: The Open Source OS for the Internet of Things. http://www.contiki-os.org/.

[41] Gleonec P.D., Ardouin J., Gautier M., Berder O. A Real-World Evaluation of Energy Budget Estimation Algorithms for Autonomous Long Range IoT Nodes. Proceedings of the IEEE International Conference on Telecommunications (ICT).

[42] Ait Aoudia F., Gautier M., Magno M., Berder O., Benini L. (2018). Leveraging Energy Harvesting and Wake-Up Receivers for Long-Term Wireless Sensor Networks. Sensors.

[43] Le Mag Big Data: 8 Projets Retenus pour Préparer la Ville de Demain. http://www.lemag-numerique.com/2015/10/big-data-8-projets-retenus-pour-preparer-la-ville-de-demain-7989.

[44] Wang W.H., Palaniswami M., Low S.H. (2006). Application-Oriented Flow Control: Fundamentals, Algorithms and Fairness. IEEE/ACM Trans. Netw..

[45] Kuhn H.W., Tucker A.W. (1951). Nonlinear Programming. Proceedings of the Second Berkeley Symposium on Mathematical Statistics and Probability.

[46] Wi6labs Wi6labs Website. http://www.wi6labs.com.

[47] ANRT Association Nationale Recherche Technologie. http://www.anrt.asso.fr/fr.

